# Rh type C-glycoprotein functions as a novel tumor suppressor gene by inhibiting tumorigenicity and metastasis in head and neck squamous cell carcinoma

**DOI:** 10.18632/aging.102000

**Published:** 2019-06-06

**Authors:** Wenguang Xu, Huihui Zou, Zheng Wei, Chuanhui Song, Chuanchao Tang, Xiteng Yin, Yufeng Wang, Shengwei Han, Yu Cai, Wei Han

**Affiliations:** 1 Department of Oral and Maxillofacial Surgery, Nanjing Stomatological Hospital, Medical School of Nanjing University, Nanjing, China; 2 Central Laboratory of Stomatology, Nanjing Stomatological Hospital, Medical School of Nanjing University, Nanjing, China

**Keywords:** HNSCC, RHCG, tumorigenicity, metastasis, aging, age-related diseases

## Abstract

Head and neck squamous cell carcinoma (HNSCC), a major histologic subtype of head and neck cancer, presents great mortality and morbidity worldwide. The aim of this study is to discover new potential biomarkers closely correlated with HNSCC progression. In this study, weighted gene co-expression network analysis was applied to construct a co-expression network, and the brown module was identified as the most correlated with HNSCC progression. Hub gene identification combined with survival analyses determined RHCG as a candidate biomarker for cancer progression and prognosis prediction. Further experimental results proved that RHCG was aberrantly downregulated in HNSCC tissues and cell lines. Moreover, decreased RHCG expression was shown to be associated with advanced stage and dismal prognosis in HNSCC patients. Functional assays revealed that RHCG could inhibit cell viability, clonogenicity, cell migration *in vitro* and suppress tumor formation *in vivo*. Further bioinformatics study demonstrated that DNA promoter hypermethylation of RHCG could lead to its downregulation and serve as potential prognostic maker in HNSCC. Our study reveals that RHCG acts as a tumor suppressor gene that plays a crucial role in inhibiting tumorigenicity and metastasis in HNSCC, which will shed light on the potential diagnostic and therapeutic strategies for HNSCC.

## INTRODUCTION

Head and neck squamous cell carcinoma (HNSCC) is the most common histologic subtype of malignancy in head and neck region. With surgical advances and escalation of treatment modalities, the 5-year overall survival rate for HNSCC patients has been increased to 50%–60%. However, for HNSCC patients with advanced stage tumors, the clinical outcome and long-time survival remains poor, remaining at 30%–40% [1, 2]. In clinical practice, clinicians usually assigned appropriate therapeutic schedules to HNSCC patients based on their clinical traits as guided by American Joint Committee on Cancer (AJCC) staging systems [3]. To date, various biomarkers have been employed in cancer studies for early detection, differential diagnosis, prediction of therapeutic response, monitoring tumor progression and prognosis evaluation [4]. Thus, further identification of novel biomarkers that are strongly associated with tumor progression and clinical outcomes of HNSCC patients might be of clinical significance.

Currently, microarrays and RNA sequencing technology have been widely used to explore candidate genes associated with HNSCC progression [5, 6]. Numerous gene expression profiles of HNSCC have been uploaded and stored in public databases, such as The Cancer Genome Atlas (TCGA), Gene Expression Omnibus (GEO) database, and ArrayExpress. However, most of the related studies have too much emphasis on the screening of differentially expressed genes neglecting of the potential interconnection between genes. Weighted gene co-expression network analysis (WGCNA), a systems biology algorithm, can be used as a data exploratory tool to explore the correlation patterns among genes across microarray data and find modules of high correlated genes and related modules to clinical features [7]. By now, WGCNA has been employed in a variety of studies to reveal phenotype-related genes and identify candidate biomarkers or therapeutic targets, especially in cancer research [8]. With the identification of gene modules or clusters that are closely related to the phenotype characteristics of samples, it may facilitate the elucidation of the potential mechanism associated with different biological processes and clinical traits.

Rh type C glycoprotein (RHCG), a member of Rhesus (Rh) family, was originally identified as Rh blood group antigens in human erythroid cells [9, 10]. It was reported to play a fundamental role in ammonium handling and pH homeostasis in the kidney [11]. In addition, RHCG has a hydrophobic ammonia conduction channel and shares a common folding structure with ammonia transporter. On account of the special structure of RHCG, it may function as an ammonia-specific transporter [12]. Accordingly, RHCG is widely expressed in differently tissues where ammonia metabolism is indispensable for exerting normal biological functions. Previous evidence has shown that RHCG is downregulated in esophageal squamous cell carcinoma and tongue squamous cell carcinoma, but expressed in multiple normal squamous epithelia [13, 14]. Recent studies revealed that RHCG could exert suppression effects on cell proliferation, migration, and invasion in esophageal squamous cell carcinoma and cervical cancer [15, 16]. However, the role of RHCG in HNSCC has not been fully understood and remained to be investigated.

In the present study, we identified RHCG as a candidate biomarker associated with HNSCC tumorigenesis and progression. RHCG downregulation was also correlated with advanced clinical stage, lymphatic metastasis, and poor prognosis of HNSCC patients. More importantly, *in vitro* and *in vivo* experiments proved that RHCG was a tumor suppressor gene in HNSCC. DNA promoter hypermethylation might contribute to RHCG inactivation in HNSCC.

## RESULTS

### Identification of clinically significant modules by weighted correlation network analysis

Firstly, HNSCC patients with full transcriptome data and complete and definite clinical features were obtained from TCGA database. After the inclusion criteria were applied, a total of 299 HNSCC patients were included in the study, comprising 299 tumor tissue samples and 16 normal tissue samples. After data preprocessing and quality assessment, the transcriptome data from 299 HNSCC tissues and 16 normal tissues were further analyzed. With the threshold of adjust p value< 0.05 and |fold change| > 2, 4563 DEGs were screened out, of which 2072 were upregulated and 2491 were downregulated in HNSCC tissues compared to normal tissues. The DEGs are shown in the volcano map, and the top 100 DEGs are also visualized on a heatmap (Supplementary Figure 1A and 1B).

**Figure 1:**
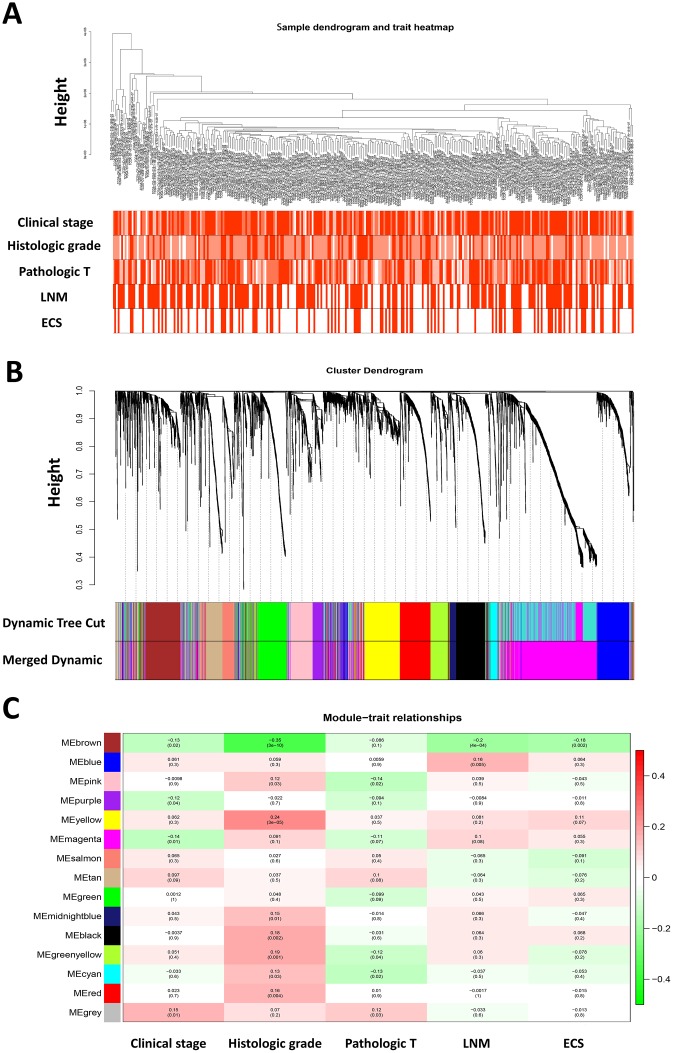
**Weighted gene co-expression network analysis and identification of modules associated with the progression of HNSCC.** (**A**) Clustering dendrogram of 299 HNSCC samples and the clinical traits. The clustering was based on the expression data of differentially expressed genes between tumor samples and normal samples in HNSCC. The color intensity was proportional to more advanced clinical stage as well as higher histologic grade and pathologic T stage. In lymph node metastasis (LNM) and nodal extracapsular spread (ECS), the red color represented pathologic nodal metastasis and nodal capsular spread. (**B**) Dendrogram of all differentially expressed genes clustered based on a dissimilarity measure (1-TOM). (**C**) Heatmap of the correlation between module eigengenes and disease progression features of HNSCC. The upper number in each cell refers to the correlation coefficient of each module in the trait, and the lower number is the corresponding p-value.

Five clinical features associated with HNSCC progression including clinical stage, histologic grade, pathologic T stage, pathologic nodal metastasis and nodal extracapsular extension of 299 HNSCC patients were included in the analysis (Figure 1A). To ensure a scale-free network, the power of β = 4 (scale free R^2^ = 0.85) was selected (Supplementary Figure 2A). After determination of the soft threshold, all of DEGs from 299 HNSCC samples were used to construct weighted gene co-expression networks Briefly, the correlation matrix and adjacency matrix of the gene expression profiles of HNSCC patients were calculated and then transformed into a topological overlap matrix (TOM). Subsequently, a system clustering tree of genes on the basis of gene-gene non-ω similarity was obtained (Supplementary Figure 2B). Together with the TOM, the hierarchical average linkage clustering method was employed to identify the gene modules of the coexpression network (Supplementary Figure 2C). With a minimum module size of 40 for the genes dendrogram and a cut-line of 0.25 for module dendrogram and merged some modules, a total of fifteen gene modules were recognized by the dynamic tree cut (Figure 1B).

It is of great clinical significance to identify modules most significantly associated with clinical features. After correlating modules to clinical traits, it was shown that Brown module was the most relevant with cancer progression traits, among which the highest association was found between Brown module and histologic grade (r = 0.35, p = 3e-10; Figure 1C). In addition, the associated Gene Significance (the correlation between the genes and the traits) and Module Membership (the correlation of the module eigengene and the gene expression profile) of fifteen modules was calculated, and it was found that genes in Brown module tended to be highly correlated with HNSCC progression, especially correlated with histologic grade and pathological nodal metastasis (Supplementary Figure 2D). Therefore, the brown module was selected to be further investigated.

### Hub genes identification reveals RHCG as a candidate biomarker in HNSCC

**Figure 2:**
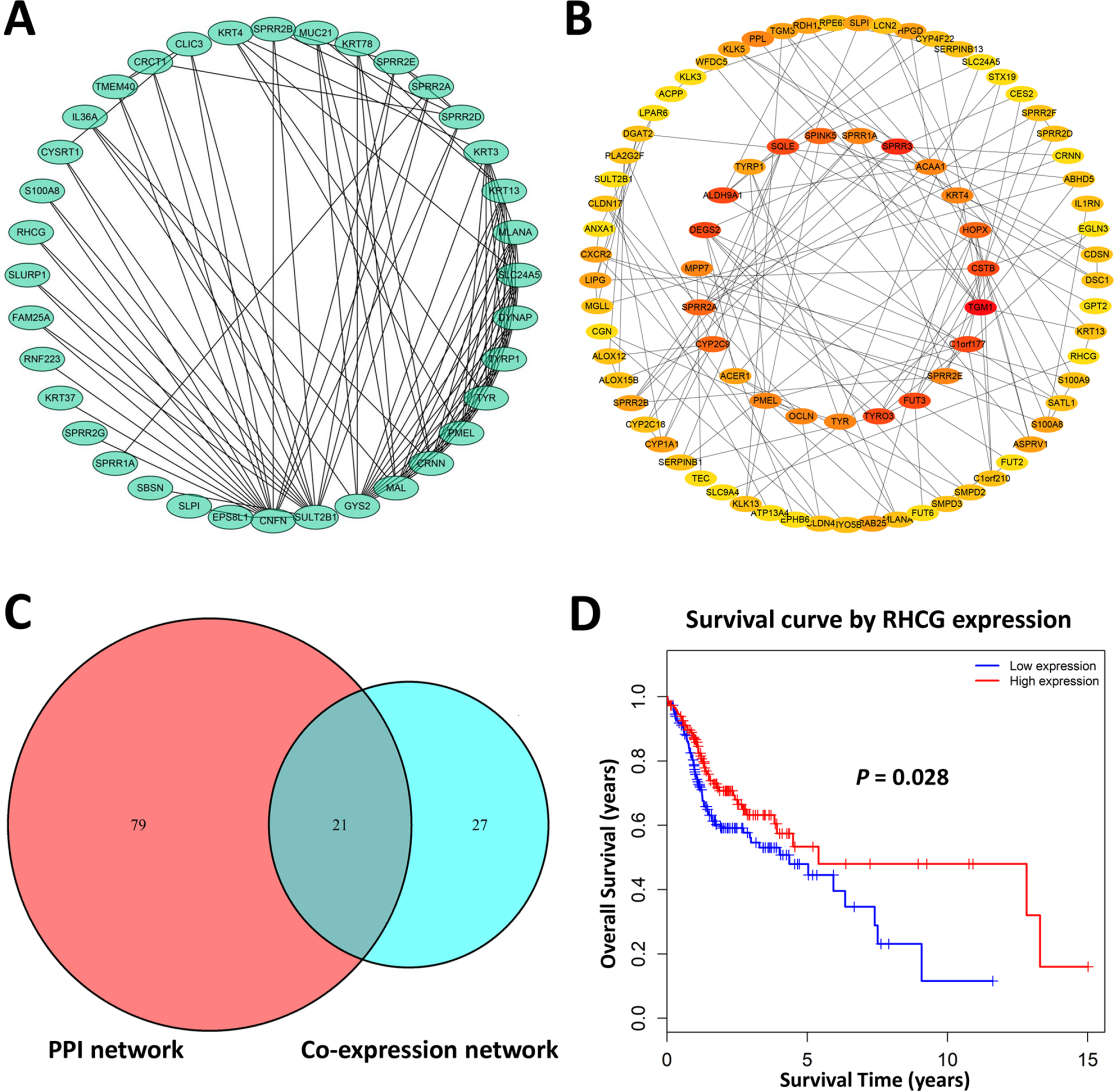
**Hub genes identification.** (**A**) Genes with a combined weight score > 0.2 in brown module were taken as hub genes in the co-expression network (**B**) Protein–protein interaction network of genes in the brown module. The color intensity in each node was proportional to the degree of connectivity in the network. (**C**) Selection of common hub genes in co-expression network and PPI network. (**D**) Survival analysis showed that RHCG exhibited the significant prognostic value for HNSCC patients.

Defined by a combined weight score > 0.2 among genes in brown module, a total of 48 genes highly connected in brown module were determined as candidate hub genes (Figure 2A). Furthermore, we constructed a protein-protein interaction network containing 178 nodes and 230 protein pairs by Cytoscape via STRING database, and top 100 high degree genes were displayed in a network (Figure 2B). Finally, 21 common genes in both networks were considered as hub genes to be further analyzed and validated (Figure 2C).

To explore the potential hub genes that might be of clinical significance for HNSCC, Kaplan-Meier survival curves of each hub gene were plotted for 299 HNSCC patients in the present cohort. As a result, only RHCG showed significant prognostic value (p<0.05). Patients with higher expression of RHCG showed significantly longer overall survival rate, indicating that RHCG might serve as a favorable prognosis biomarker of HNSCC (Figure 2D). Hub genes that did not harbor prognostic value were exhibited in Supplementary Figure 3. Overall, RHCG was identified as a pivotal candidate gene due to its key role in the disease progression and significant prognostic value in HNSCC.

### RHCG is aberrantly downregulated in HNSCC tissues and cell lines

**Figure 3:**
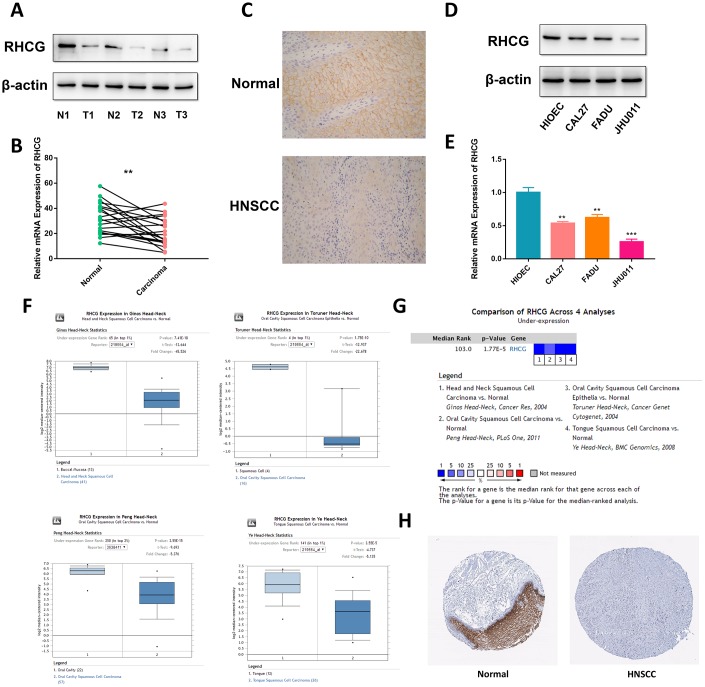
**RHCG is downregulated in HNSCC tissues and cell lines.** (**A**) Western blotting analysis of RHCG protein levels in HNSCC and normal tissues (n=3). (**B**) mRNA expression of RHCG in HNSCC and corresponding normal tissues (n=20). (**C**) Expression of RHCG as examined by immunohistochemistry (×400) in in HNSCC and normal tissues. (**D**) Western blotting analysis of RHCG expression in a human immortalized oral epithelial cell (HIOEC) line and HNSCC cell lines. (**E**) Real-time PCR analysis of relative RHCG mRNA expression levels HIOEC cells and HNSCC cell lines. (**F**) Comparison of RHCG expression in cancerous tissues and normal tissues in 4 different cohorts, including Ginos Head-Neck, Peng Head-Neck, Toruner Head-Neck and Ye Head-Neck according to the Oncomine database. (**G**) Meta-analysis of RHCG expression across the 4 analyses. (**H**) RHCG protein expressed in normal and HNSCC cancerous tissues from The Human Protein Atlas database. The tissue expressing abundant RHCG signals was normal tongue tissue from a female at an age of 73. RHCG was strongly stained in squamous epithelial cells, mainly located in cytoplasmic or membranous region; the tissue with no staining was head and neck squamous cell carcinoma tissue from an 81-year-old woman.

To confirm the above findings, RCHG expression was analyzed in HNSCC and adjacent normal tissues. Western blotting and real-time quantitative PCR results confirmed that RHCG was downregulated in neoplastic tissues compared to normal tissues (Figure 3A and 3B). Moreover, representative hematoxylin and eosin (H&E) staining in HNSCC tissues and immunohistochemistry (IHC) confirmed these results (Figure 3C). In addition, the expression of RHCG was evaluated in a panel of HNSCC cell lines (CAL27, FADU and JHU011), with HIOEC cells serving as a normal control. It was shown that RHCG protein was significantly decreased in HNSCC cell lines in comparison with HIOEC cells (Figure 3D), which was consistent with the results of real-time quantitative PCR (Figure 3E). Besides, based on the Oncomine database, it was noteworthy that the expression of RHCG was significantly reduced in HNSCC tissues compared with normal tissues with profiling data obtained from four different studies (Figure 3F). The meta-analysis of the expression data across the four studies further validated the above results (Figure 3G). A pan-cancer study of RHCG expression pattern based on database revealed that RHCG was downregulated in multiple cancers, with HNSCC listed as the most significantly downregulated (Supplementary Figure 4A). Importantly, through immunohistochemistry staining data obtained from The Human Protein Atlas database, it was observed that RHCG was strongly expressed in normal tissues in head and neck region like oral mucosa, tonsil and salivary gland, while most of the tumor tissues exhibited low expression of RHCG or negative (Figure 3H, Supplementary Figure 4B).

**Figure 4:**
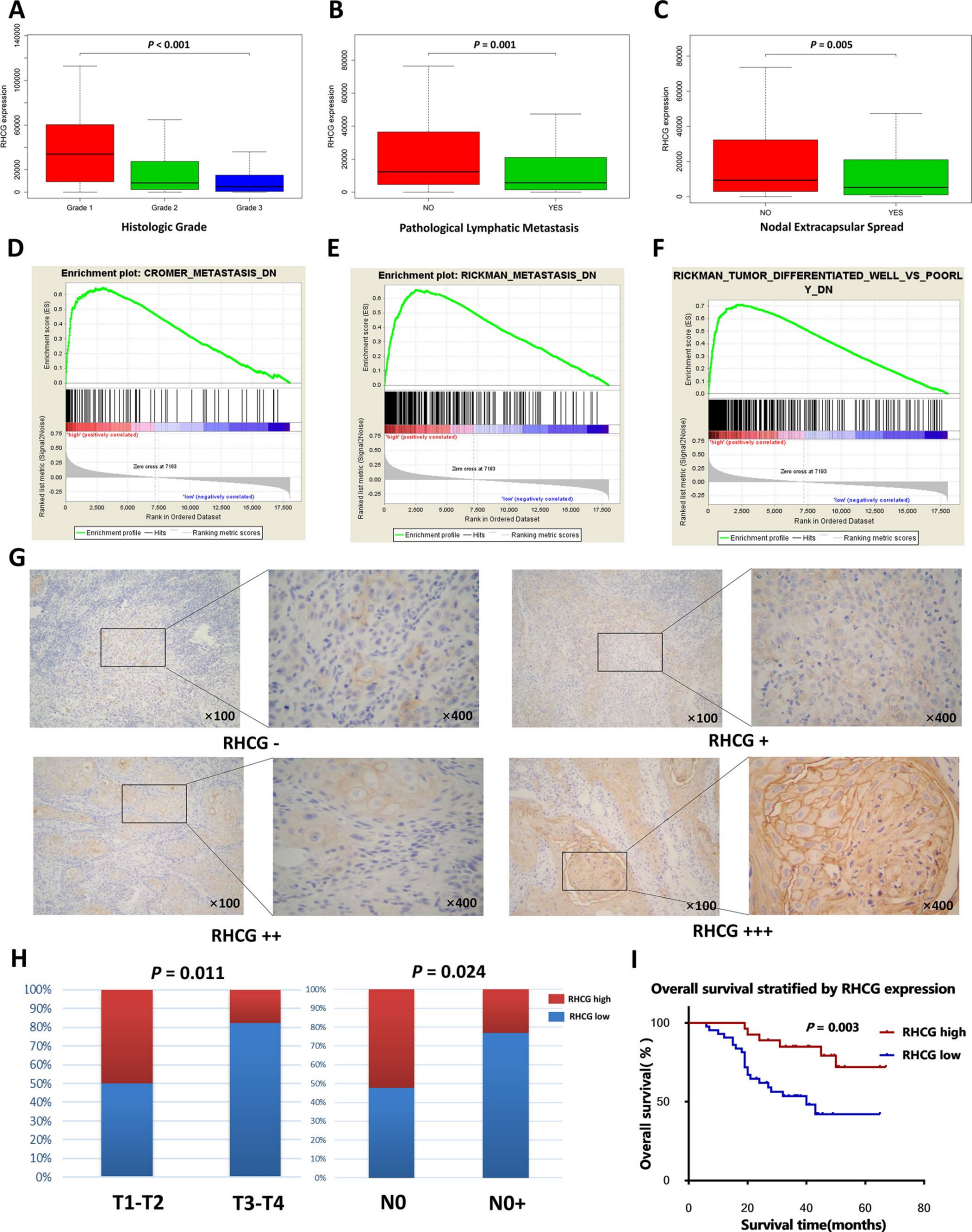
**RHCG is correlated with HNSCC progression-associated traits.** (**A**–**C**) The expression levels of RHCG in different subgroups of 299 HNSCC patients stratified by histologic grade (**A**), pathologic nodal metastasis(B) and nodal extracapsular spread in the TCGA database; (**D**–**F**). GESA analysis of HNSCC-specific tumor progression signatures enriched in RHCG-high or RHCG-low HNSCC patients in the TCGA database. (**G**) Representative immunohistochemical staining degrees for RHCG in 70 HNSCC tissues. (**H**) Correlation of RHCG expression with tumor stage and lymph node metastasis in 70 HNSCC patients by Fisher's exact test. (**H**) Kaplan–Meier curves of 70 HNSCC patients stratified by the RHCG expression, log-rank test was used to test the difference between two groups.

### Low RHCG expression is associated with advanced stage and poor prognosis in HNSCC patients

To further investigate the role of RHCG in the progression of HNSCC, Kolmogorov-Smirnov test was employed to compare RHCG expression across different groups of HNSCC patients. It was striking that RHCG was significantly downregulated in patients with higher histologic grade, presence of pathologic nodal metastasis and nodal extracapsular spread (p<0.05) (Figure 4A–4C). Moreover, GSEA was conducted to explore relevant HNSCC progression-associated gene signatures based on the expression of RHCG. Strikingly, low expression of RHCG was correlated with previously established HNSCC-specific progressive signatures such as “CROMER metastasis up”, “RICKMAN metastasis up” and “RICKMAN tumor differentiated well vs poorly up” (Figure 4D–4F). To further verify the relationship between RHCG expression and clinical features, IHC assays were performed in a validation cohort of 70 HNSCC patients from our institution (Figure 4G). It was shown that proportion of HNSCC patients with RHCG low expression was significantly increased in HNSCC patients with a more advanced tumor stage and lymph node metastasis (Figure 4H). Moreover, the Kaplan–Meier analysis revealed that low RHCG expression was associated with an unfavorable prognosis by log-rank test (Figure 4I). Univariate and multivariate analysis also identified RHCG as an independent prognostic factor in HNSCC (Table 1).

**Table 1:** Univariate and multivariate survival analysis for patients with HNSCC.

**Variable**	**Univariate survival analysis**		***P*- value**	**Multivariate survival analysis**		***P*- value**
	**Hazard ratio**	**95%CI**		**Hazard ratio**	**95%CI**	
Age	2.036	0.937–4.424	0.072			
(≥65, < 65)						
Gender	1.445	0.678–3.125	0.336			
(male, female)						
T stage	2.647	1.237–5.665	**0.012**			
(T1-T2, T3-T4)						
N stage	2.232	1.471–3.387	**0.000**	1.900	1.230–2.937	**0.004**
(N0, N1, N2)						
Histologic stage	1.377	0.719–2.637	0.334			
(I, II, III)						
Subdivision	0.993	0.761–1.295	0.958			
(tongue, buccal, palate, gingiva)						
RHCG expression	0.163	0.157–0.473	**0.001**	0.354	0.134–0.932	**0.036**
(low, high)						

The number in bold indicate statistical significance.

### RHCG attenuates HNSCC cell proliferation and migration

**Figure 5:**
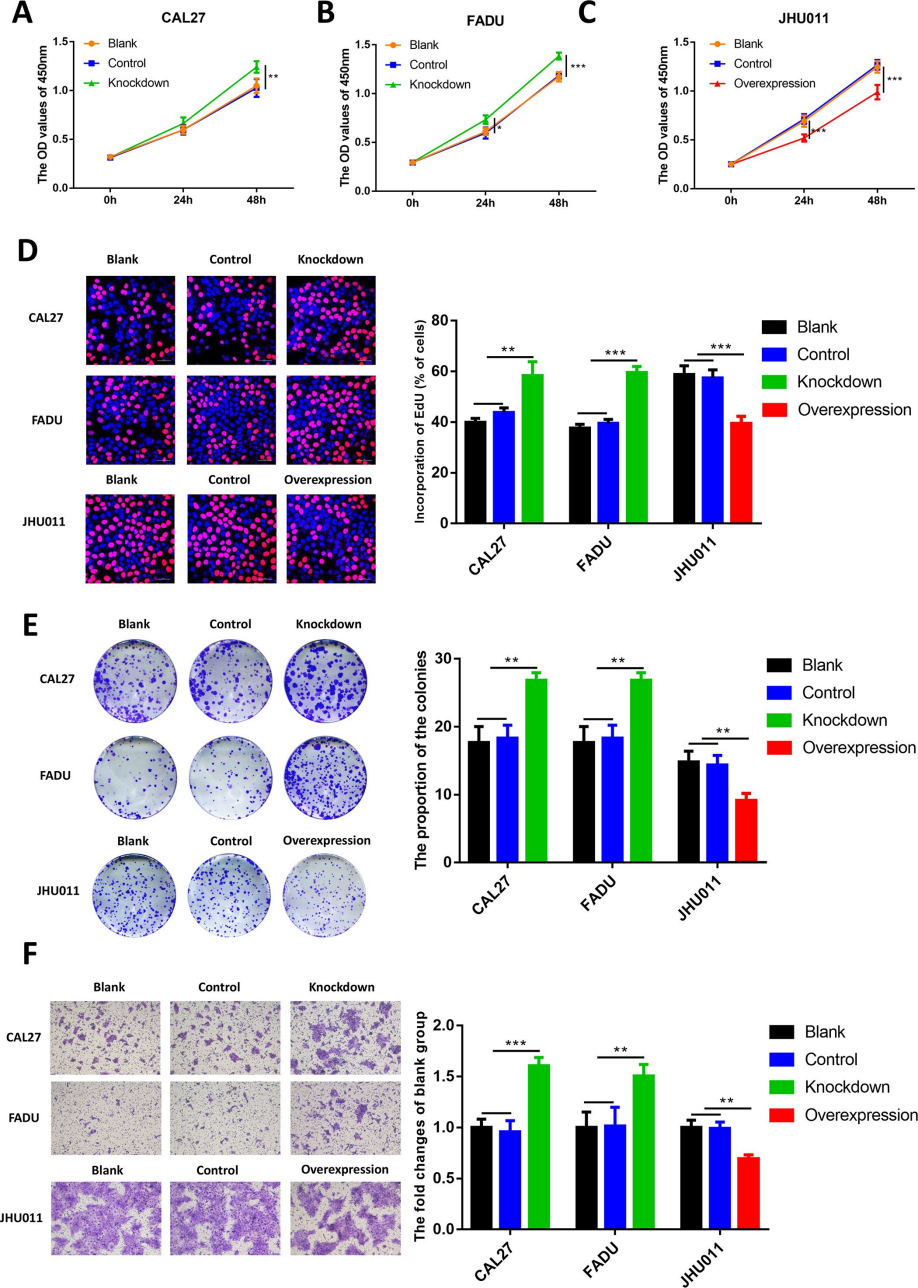
**Effects of RHCG on HNSCC cell proliferation, colony formation and migration.** (**A**–**C**) Cell growth of CAL27 and FADU cells after RHCG knockdown and JHU011 cells after RHCG overexpression as determined by CCK8 assay at different time points. (**D**) The proliferation of CAL27 and FADU cells after RHCG knockdown and JHU011 cells after RHCG overexpression was evaluated by EdU assay. Representative images for EdU-positive cells (red) and Hoechst-stained nuclei (blue) are shown. Scale bars represent 50 μm. Quantification of the percentage of EdU-positive cells is shown on the right. (**E**) Colony-forming growth assay was performed to determine the proliferation of CAL27 and FADU after RHCG knockdown and JHU011 cells after RHCG overexpression. Colonies were counted and calculated on the right. **P < 0.01 compared with the NC group. (**F**) Transwell migration assay of migratory capacity of CAL27 and FADU after RHCG knockdown and JHU011 cells after RHCG overexpression. Data are presented as the mean ± SD of three independent experiments on the right. *P < 0.05, **P < 0.01, compared with the NC group.

To further investigate the role of RHCG in HNSCC cells, CCK8 assay was performed. Results revealed that knockdown of RHCG dramatically increased HNSCC cell proliferation in comparison with the control group (Figure 5A and 5B). Diametrically, overexpression of RHCG in JHU011 cells lead to attenuated cell proliferation (Figure 5C). In addition, ethynyl deoxy uridine (EdU) staining assays were applied to confirm the effect of RHCG on the proliferative ability of HNSCC cells. It was shown that proliferative rate of CAL27 and FADU cells treated with RHCG-shRNA was remarkably increased compared with negative control, while overexpression of RHCG in JHU011 cells showed the opposite effect (Figure 5D). Consistent with the results of CCK-8 assays and EdU assays, colony formation assay showed that knockdown of RHCG could promote HNSCC cell proliferation, whereas overexpression of RHCG promoted cell proliferation (Figure 5E). We then measured the influence of RHCG on cell migration using Transwell assay. The results demonstrated that knockdown of RHCG significantly boosted cell migration ability while overexpression of RHCG alleviated the migration (Figure 5F). Taken together, these results indicated that RHCG suppressed HNSCC cell proliferation and migration.

### RHCG inhibits HNSCC cell tumorigenesis *in vivo*

**Figure 6:**
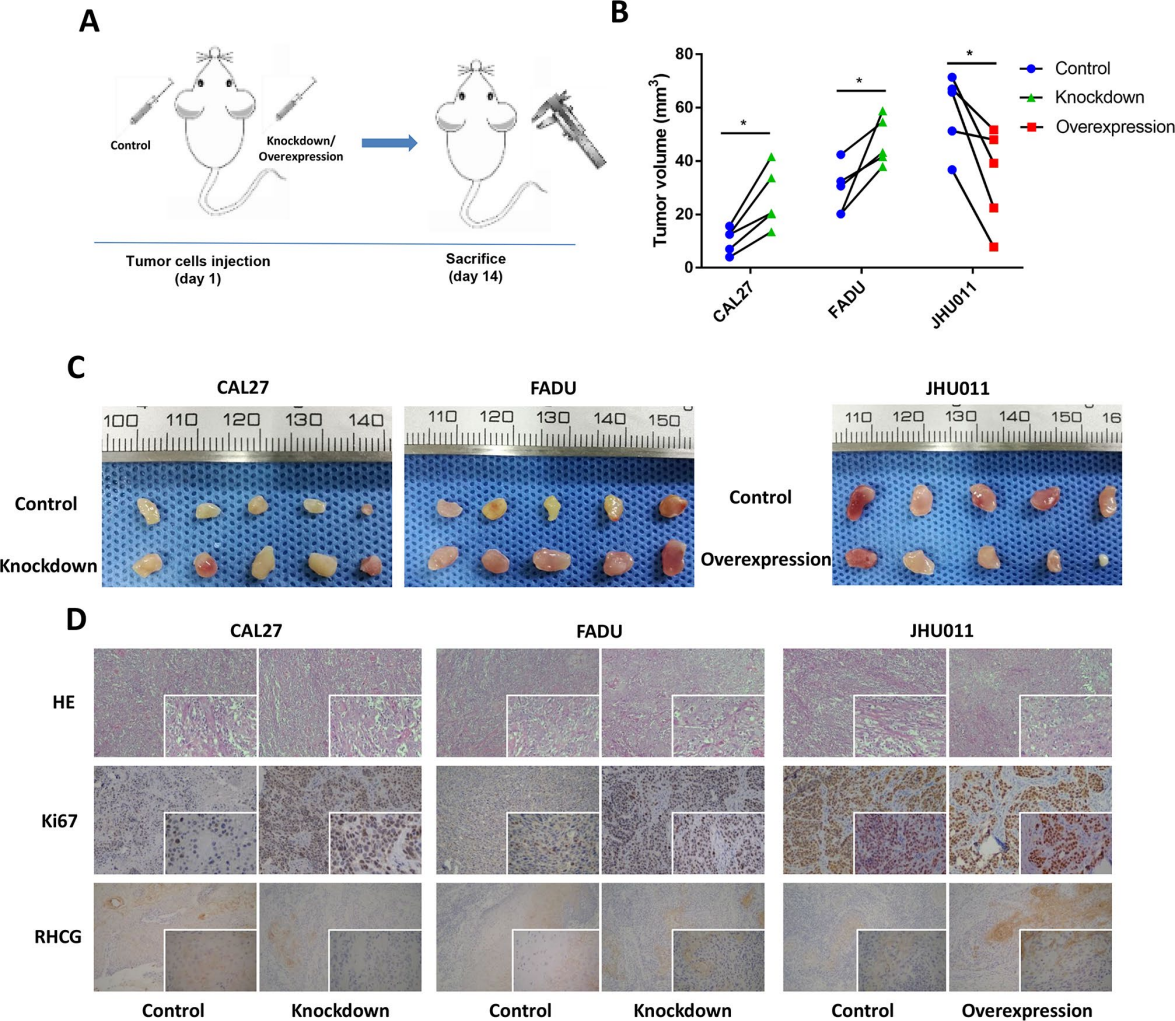
**Effects of RHCG on tumor growth *in vivo*.** (**A**) Diagram of *in vivo* assay procedure; (**B**) Tumor volumes, measured at experiment endpoints in in control and RHCG knockdown or overexpression xenografts (n = 5 for each group). (**C**) Tumor images at experimental endpoints in control and RHCG knockdown or overexpression xenografts (n = 5 for each group). (**D**) The tumor sections were under H&E staining and IHC staining using antibodies against ki-67 and RHCG. Bar graphs (mean ± SD) and representative images are shown. *P < 0.05, **P < 0.01, compared with the NC group.

To confirm whether RHCG influences HNSCC tumorigenesis *in vivo*, nude mice received subcutaneous injections of RHCG-knockdown and RCCG-overexpression cells to establish the xenograft model (Figure 6A). At 14 days post-injection, the volumes of tumors established in the RHCG-knockdown group (CAL27 and FADU) were dramatically bigger than those in the control group. Conversely, the tumor volume of RHCG overexpression group was much smaller than NC group (Figure 6B and 6C).

Furthermore, the effects of RHCG inhibition on HNSCC growth were supported by the lower protein levels of Ki67 as determined by immunohistochemistry (Figure 6D). Together, these results indicated that RHCG suppressed HNSCC growth *in vivo*.

### DNA promoter hypermethylation of RHCG may lead to its downregulation in HNSCC

**Figure 7:**
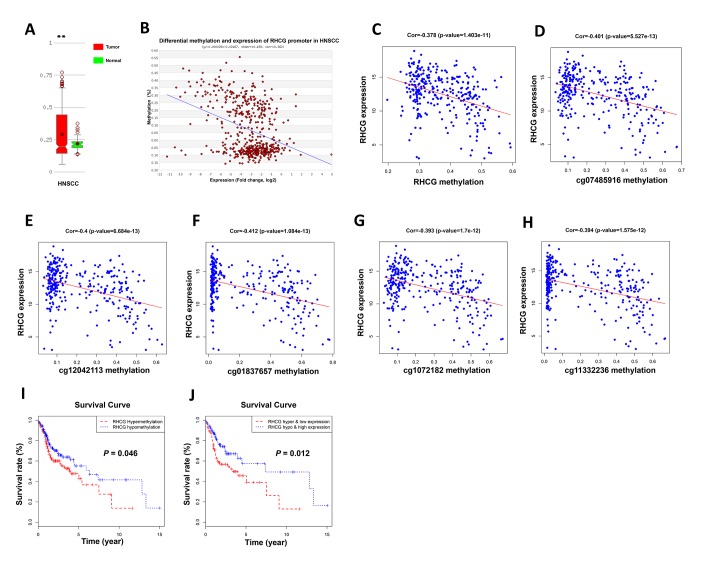
**Hypermethylation of RHCG promoter region lead to downregulation of RHCG.** (**A**) Comparison of methylation level of RHCG in HNSCC samples and normal tissues based on MethHC database. (**B**) Correlation between RHCG expression and RHCG promoter methylation according to MethHC database. (**C**) Correlation between RHCG expression and RHCG methylation in the present cohort of 299 HNSCC patients. (**D**–**H**) Correlation between RHCG expression and five methylation sites in RHCG promoter region of 299 HNSCC patients. (**I**–**J**) Survival curves of 299 HNSCC patients stratified by RHCG methylation level (**I**) and RHCG methylation and expression level (**J**).

Since DNA promoter hypermethylation is commonly implicated in inactivation of tumor suppressor genes in cancers, we then investigated whether the RHCG promoter hypermethylation may impair RHCG expression in HNSCC. Through annotation of RHCG in UCSC database, it was noteworthy that there existed CpG islands in gene region RHCG (Supplementary Figure 5). We next considered whether RHCG methylation might attribute to downregulation of RHCG in HNSCC. With data from MethHC database, it was evident that the overall methylation level of RHCG was higher in HNSCC tissues than that in normal tissues (p < 0.05) (Figure 7A). Moreover, the expression of RHCG was negatively correlated with RHCG promoter methylation level in HNSCC (r = −0.362, p < 0.05) (Figure 7B). For the present cohort of 299 HNSCC patients in this study, it was also verified that there was a negative correlation between RHCG expression and RHCG methylation (r = −0.378, p < 0.05) (Figure 7C). To be more specific, the correlation between RHCG expression and five methylation sites in the promoter of RHCG was calculated. Strikingly, a negative correlation was found between RHCG expression and methylation level of each methylation site in the promoter region (Figure 7D–7H). To determine the prognostic potential of RHCG methylation, the survival curve of 299 HNSCC patients stratified by RHCG methylation level was plotted, it was evident that patients with RHCG hypermethylation had a significantly shorter overall survival time compared with patients with RHCG hypomethylation (p=0.046) (Figure 7I). Furthermore, patients with RHCG hypermethylation and RHCG low expression demonstrated a significantly shorter overall survival compared to those with RHCG hypomethylation and RHCG high expression (p = 0.012) (Figure 7J). By combining RHCG methylation level and RHCG expression level, it exhibited a much better prognostic capacity to distinguish patients with poor prognosis from those with good prognosis. Together, these results indicated that DNA promoter hypermethylation was implicated in RHCG inactivation in HNSCC. More importantly, methylation status of RHCG could serve as a potential prognostic maker for HNSCC.

## DISCUSSION

HNSCC is the most common subtype among head and neck cancers. Exploring molecular markers correlated with HNSCC progression is crucial for improving risk stratification, therapeutic decision-making and prognosis prediction in patients [17, 18]. As a systematic biological approach, WHCNA provided a global interpretation of gene expression. It has been widely utilized to identify candidate biomarkers and biological pathways related to disease initiation and progression in multiple cancers [19–22]. Herein, WGCNA was employed to determine gene co-expression modules associated with progression of HNSCC. In our study, the brown module was identified and 21 common hub genes were derived from the module both in co-expression network and PPI network. After survival analyses of hub genes, RHCG was finally identified as a candidate biomarker, which was not only highly correlated with HNSCC progression, but also might serve as a potential prognosticator.

RHCG is a relatively conserved gene that encodes a plasma membrane protein sharing strong similarity to Rh proteins in amino acid sequence. Recently, a minority of studies have reported that RHCG was frequently downregulated in esophageal and tongue squamous cell carcinomas and cervical cancer, while strongly expressed in multiple squamous epithelia [13–16]. Based on the GEIPIA database, we also found that RHCG was downregulated in esophageal carcinoma, glioblastoma multiforme, head and neck squamous cell carcinoma, kidney renal clear cell and papillary cell carcinoma, brain lower grade glioma and so on, while upregulated in kidney chromophobe and lung squamous cell carcinoma.

In the present study, RHCG was identified to be significantly downregulated in HNSCC tissues and correlated with tumor progression based on TCGA dataset. To further verify the results, the Oncomine database and The Human Protein Atlas were used. In addition, RHCG downregulation was observed in HNSCC cell lines and neoplastic samples based on qPCR assay and Western blotting. Importantly, it was found that RHCG downregulation was associated with higher histologic grade, pathologic nodal metastasis and nodal extracapsular spread. The gene set enrichment analysis revealed that low expression of RHCG was correlated with previously established HNSCC-specific progressive signatures. In a validation cohort from our institution, RHCG was further validated to be associated with lymph node metastasis and an unfavorable prognosis. These results provided strong evidence for the role of RHCG in HNSCC.

To provide abundance evidence for the tumor inhibitory effect of RHCG in HNSCC, both *in vitro* and *in vivo* functional assays were performed. RHCG silencing in HNSCC cell lines CAL27 and FADU could effectively promote cell viability, colony formation and cell migration and tumor formation in nude mice, while ectopic expression of RHCG in JHU011 cells could suppress these functions, which was in consistence with previous studies [15, 16]. In addition, the molecular mechanisms underlying the tumor suppressive functions of RHCG have been previously investigated. Ming et al reported that RHCG could suppress the tumorigenicity and metastasis of esophageal squamous cell carcinoma via inhibiting NF-κB signaling and MMP1 expression [15]; Wang et al discovered that RHCG might suppress cervical cancer progression through inducing apoptosis regulated by TGF-β1 [16]. In the present study, we conducted a pilot bioinformatics study by gene set enrichment analysis, it was suggested that RHCG might be involved in potential pathways such as “ECM receptor interaction”, “Focal adhesion”, “alpha linolenic acid metabolism” and “arachidonic acid metabolism”, however, further validation was still needed (Supplementary Figure 6).

Downregulation of tumor suppressor genes is commonly observed in cancers, which is often caused by promoter hypermethylation, histone deacetylation, dysregulation by transcription factors and posttranscriptional silencing by microRNAs [23]. In the present study, through further bioinformatics analysis, it was also shown that RHCG promoter hypermethylation might impair RHCG expression. More importantly, methylation status of RHCG exhibited a promising prognostic potential for HNSCC. Strand et al. also found that RHCG promoter hypermethylation could predict biochemical recurrence in prostate cancer patients, adding significant independent prognostic value to established prognostic parameters [24].

In summary, our study identified RHCG as a candidate biomarker correlated with HNSCC tumor progression and dismal prognosis, which acted as a tumor suppressor gene by playing a pivotal role in impeding tumorigenicity and metastasis in HNSCC. RHCG promoter hypermethylation might lead to RHCG inactivation and serve as potential prognostic maker in HNSCC.

## MATERIALS AND METHODS

### TCGA datasets and differentially expressed genes (DEGs) screening

RNA sequencing data and clinical information of 528 HNSCC patients was downloaded from The Cancer Genome Atlas (TCGA) database (https://cancergenome.nih.gov/). The sequenced data were derived from Illumina HiSeq RNASeq platform. Selection criteria of the samples were: (1) they were HNSCC tissues; (2) clinical information of the patients was provided including neoplasm subdivision, clinical stage, histologic grade, pathologic T stage, pathologic N stage and presence of nodal extracapsular spread. Data processing procedures met the requirements of the data access policies and NIH TCGA human subject protection (http://cancergenome.nih.gov/publications/publicationguidelines). The “limma” R package [25] was used to identify the DEGs between HNSCC tissues and normal tissues. The criteria with FDR (false discovery rate) < 0.05 and |log2 fold change (FC)| > 1 were applied to determine genes for further construction of co-expression network.

### Co-expression network construction and clinically significant modules identification

The “WGCNA” package [7] in R was employed to establish co-expression network by using the expression values of DEGs from 299 tumor samples and complete clinical data including clinical stage, histologic grade, pathologic T stage, pathologic nodal metastasis and nodal extracapsular extension. Firstly, the hierarchical clustering was used to perform the preliminary classification of 299 patients by average-linkage agglomerative algorithm. Then, we constructed the weighted adjacency matrix using a power function based on a soft-thresholding parameter β. Subsequently, the adjacency was transformed into topological overlap matrix (TOM), and average linkage hierarchical clustering was performed according to the TOM-based dissimilarity measure. In the study, a minimum size (gene group) of 40 for the genes dendrogram and a cut-line (0.25) for module dendrogram and merged some modules was determined. Gene modules were identified based on their expression similarities in samples. The correlation between the clinical traits and gene modules were calculated to determine clinically significant gene modules. The gene modules most associated with clinical features were selected as modules of interest.

### Hub genes identification and survival analysis

In co-expression network, hub genes were defined by a combined weight score > 0.2. In addition, we used Search Tool for the Retrieval of Interacting Genes (STRING) database (http://www.string-db.org/) combined with Cytoscape software to establish protein-protein interaction (PPI) network. Confidence score > 0.4 was set as significant. In order to identify the mRNAs that harbor prognostic value, HNSCC samples were divided into two groups based on the median expression value of each gene in the present cohort, and survival curves were depicted using “survival” package in R software. The log rank test was used to compare the survival difference between two groups. The P-value of 0.05 was considered as statistically significant.

### Gene set enrichment analysis (GSEA)

A total of 299 HNSCC samples containing RNA-sequecing data were divided into two groups (high vs. low) based on the expression level of RHCG and median expression value was used as the cut-off point. In order to figure out potential function and signatures correlated with RHCG, GSEA was performed between the two groups. Annotated gene sets including “CROMER_METASTASIS_DN” [26], “RICKMAN_METASTASIS_DN” and “RICKMAN_TUMOR_DIFFERENTIATED_ WELL_VS_POORLY_DN” [27] were obtained from the Molecular Signatures Database as the reference gene sets. The FDR < 0.05 was set as the cut-off criteria.

### Tissue collection

All HNSCC samples, including cancerous tissues and adjacent normal tissues were obtained from the tumor biobank of Nanjing Stomatological Hospital. Briefly, the samples were derived from the HNSCC patients who underwent surgical resection at the Nanjing Stomatological Hospital. After tumor resection, the specimens were instantly frozen in tubes, kept in liquid nitrogen and stored at −80 °C until analysis. In addition, paraffin-embedded specimens of HNSCC patients were also collected and used for further HE staining and immunohistochemistry assay. The study was permitted by the Ethics Committee of Nanjing Medical University (approval number: 2019NL-001(KS)), and written informed consent was obtained from every patient.

### Cell culture and lentiviral infection

The human HNSCC cell lines CAL27 and FADU, and human immortalized oral epithelial cell (HIOEC) lines were obtained from the Cell Bank of the Chinese Academy of Sciences (Shanghai, China); JHU011 was from Johns Hopkins University (Baltimore, MD, USA). All the cell lines were cultured in DMEM (with 100 μg/mL streptomycin and 100 U/mL penicillin) (KeyGEN Biotech, Nanjing, China) supplemented with 10% FBS (BI, Migdal Haemek, Israel), in a humidified environment with 5%CO_2_. Confluent cells were trypsinzed with 0.05% trypsin containing 0.02% EDTA (Thermo Fisher Scientific, Waltham, MA, USA).

The protein and mRNA levels of RHCG in human HNSCC cell lines, CAL27, FADU and JHU011 was detected (Supplementary Figure 7A). Based on the expression levels of RHCG among HNSCC cell lines, CAL27 and FAUD cells were used for RHCG knockdown and JHU011 cells were used for RHCG overexpression.

For the knockdown of RHCG, lentiviruses carrying shRNA targeting human RHCG vectors (GeneChem, Shanghai, China) were used to infect the CAL27 and FADU cells according to the manufacturer's protocol. The shRNA sequences were listed in Supplementary Table 1. For the overexpression of RHCG, lentiviruses carrying overexpression elements (GV492) were used to infect the JHU011 cells according to the manufacturer's protocol (GeneChem, Shanghai, China). The lentiviruses carrying empty vectors were used as a negative control. The transfection efficiency was observed under a fluorescence microscope (Supplementary Figure 7B). The clones stably knocking down and overexpressing RHCG were verified by western blotting and RT-PCR (Supplementary Figure 7C–7E).

### Quantitative real-time PCR assay

Total RNA was isolated from clinical samples of HNSCC patients and HNSCC cells using TRIzol reagent. Reverse transcription was performed using a PrimeScript™ RT Reagent Kit with gDNA Eraser (Takara Biotechnology, Dalian, China) in accordance with the manufacturer’s instructions. RT-PCR was performed on ViiA™ 7 (Thermo Fisher Scientific) using the ChamQ Universal SYBR qPCR Master Mix (Vazyme Biotech, Nanjing, China) according to the manufacturer’s protocol. β-actin was used as the reference gene, and results were expressed as the relative expression ratio of target gene to reference gene. Data were analyzed with the 2−ΔΔCt method. The sequences for the primers used are listed as follows: RHCG forward, 5′-GATTTATGGTCTCTTGGTGACCCTG-3′, reverse, 5′-CTAGCTAGGTCAGCACCAGCTC-3′; β-actin forward, 5′-GCACCGTCAAGGCTGAGAAC-3′, reverse, 5′-AGCACTGTGTTGGCGTACAG-3′.

### Western blot assay

HNSCC cells were lysed on ice using the mammalian protein extraction reagent RIPA (Beyotime, Shanghai, China) with a protease inhibitor cocktail (Hoffman-La Roche Ltd, Basel, Switzerland) and phenylmethylsulphonyl fluoride (Beyotime). The supernatants were collected by centrifugation at 12,000×g at 4°C for 25 minutes. Total protein concentrations were detected by a bicinchoninic acid protein assay kit (KeyGEN Biotech). Protein samples were mixed with 5×loading buffer (GenScript, Nanjing, China) and heated at 95°C for 10 minutes. Equal amounts of protein were separated by SDS-PAGE, transferred to a 0.22 mm nitrocellulose membrane (EMD Millipore, Billerica, MA, USA) and blocked by incubation with 5% fat-free milk in TBST buffer (150 mM NaCl, 50 mM Tris-HCl, 0.5% Tween 20, pH 7.6) at room temperature for 2 hours. The membranes were incubated with primary antibodies at 4°C overnight and then with horseradish peroxidase (HRP)-conjugated secondary antibodies at room temperature for 2 hours before exposed with ECL reagent (EMD Millipore). The pictures were captured by a Tanon 6200 Luminescent Imaging Workstation (Tanon, Shanghai, China). The following primary antibodies were used to detect proteins: rabbit anti-RHCG (1:1000; Proteintech, Wuhan, China) and anti-β-actin (1:4000; Proteintech, Wuhan, China).

### Immunohistochemistry assay

Neoplastic samples and normal tissues from HNSCC patients and nude mice animal models were collected. Tissue sections (4 μm thick) were obtained, deparaffinized, and subjected to antigen recovery treatment with 100 mM citrate buffer target retrieval solution, pH 6.0 at 95°C, in a water bath for 20 minutes. Endogenous peroxidase activity was blocked by incubating with PBS and 3% hydrogen peroxidase for 30 minutes. The sections were washed by PBS and incubated with rabbit anti-RHCG (1:750; Proteintech, Wuhan, China) and Ki67 (Typing, Nanjing, China) overnight at 4°C, followed by the Envision Dual Link System HRP method (Dako Denmark A/S, Glostrup, Denmark). All the antibodies were diluted in Dako antibody diluent. Three pathologists independently scored the immunohistochemically stained slides. The scoring was based on the extent (E) of staining (percentage of positive tumor cells graded on a scale from 0 to 3: 0, none; 1, 1%–25%; 2, 26%–50%; 3, 51%–75%; 4, 75%–100%) and the intensity (I) of staining (graded on a scale of 0–3: 0, none; 1, weak staining; 2, moderate staining; 3, strong staining). The final scores were calculated using the formula: scores = ∑(E×I).

### Cell viability assay

The cell viability of HNSCC cells following RHCG knockdown and overexpression was detected by using cell counting kit-8 (CCK-8) according to the manufacturer’s instructions (Bimake, Houston, TX, USA). In brief, HNSCC cells were plated in 96-well plates with a density of 3,000 cells in 100 μL complete culture medium. The microplate was incubated overnight at 37°C until cells adhered to the plates. CCK-8 (10 μL) then was added to each well. The OD of formazan at 450 nm was recorded every 30 minutes until the OD reached 1.0–2.0. Six wells corresponded to each concentration of the abovementioned drugs. Cell viability was calculated as follows: cell viability = ([OD] test − [OD] control)/([OD] control − [OD] blank) ×100%. The IC50 value was calculated using GraphPad Prism 7 (GraphPad Software Inc, La Jolla, CA, USA).

For colony formation assay, 500 transfected HNSCC cells were combined in a six-well plate and kept in medium with 10% FBS for 2 weeks, with the replacement of medium every 4 days. Then, the colonies were confirmed with methanol and dyed with 0.1% crystal violet (SigmaAldrich) for 15 minutes. Colony formation was determined as the quantity of visibly stained colonies. Wells were measured in triplicate for the different treatment groups.

### EdU proliferation assay

HNSCC Cells were seeded in 96-well plates. After 48 h of culture, cells were incubated with EdU for 2 hours before fixation and permeabilization, and EdU staining was performed according to the manufacturer’s protocol of Cell-Light EdU Apollo567 In Vitro Kit (Ribobio, Guangzhou, China). The cell nuclei were stained with DAPI at a concentration of 1 μg/mL for 10 minutes. The proportion of cells incorporating EdU was tested through fluorescence microscopy.

### Transwell migration assay

After lentiviral transfection, the cell migration assay was performed. Briefly, 1× 10^5^ cells in 200 μl serum-free medium and complete medium was added to the lower chamber. After 24 hours, cells on the upper surface of the membrane were completely removed. Then, the cells on the lower surface of the membrane were fixed with 4% paraformaldehyde (Boster, China) for 20 minutes and stained with Crystal Violet (Beyotime, China) for 15 minutes. The cells were observed and counted under an optical microscope in five random visual fields.

### *In vivo* assay

All the animals used in this study were purchased from the Nanjing Biomedical Research Institute of Nanjing University (Nanjing, China). The nude mice (BALB/cJNju-Foxn1nu/Nju, Nanjing Biomedical Research Institute of Nanjing University, Nanjing, China) were maintained under specific pathogen-free conditions (n=30, five mice per group). RHCG knockdown or overexpressed HNSCC cells along with control group were harvested from cell culture plates, cleaned with PBS, and then resuspended at a density of 2×10^7^ cells/ml. Then, the cells were xenografted into the flank of nude mice. At 14 days post-injection, the mice were sacrificed and the xenograft tumors were removed. The sizes of the tumors were measured and then calculated. The removed tumors were fixed in formalin, paraffin-embedded, and hematoxylin and eosin staining was performed. Immunostaining analysis for RHCG and Ki67 protein expression was also carried out. All animal procedures were performed in strict accordance with the Guidelines for Care and Use of Laboratory Animals of the Medical School of Nanjing University and were approved by the Animal Ethics Committee of Nanjing Stomatological Hospital.

### RHCG methylation and expression data analysis

Briefly, MethyHC database (http://methhc.mbc.nctu.edu.tw) was initially used to compare the RHCG methylation level between HNSCC cancerous and normal tissues [28]. The correlation of RHCG methylation and RHCG expression was then analyzed. Subsequently, DNA methylation data of RHCG and survival data of 299 HNSCC patients downloaded from TCGA data portal. Correlation analysis was performed to explore if RHCG expression was affected by specific DNA methylation sites of RHCG. Finally, Kaplan-Meier survival curves were plotted and compared among subgroups (RHCG methylation high vs RHCG methylation low, RHCG methylation high & RHCG low vs RHCG methylation low & RHCG high) using log-rank tests.

### Statistical analyses

Continuous data are presented as mean ± SD. Statistical differences were measured using an unpaired two-sided Student’s t-test or one-way ANOVA for multiple comparisons when appropriate. Fisher’s exact test was used to analyze the association of RHCG expression with clinicopathologic parameters. Univariable and multivariable Cox proportional hazard regression models were used to analyze independent prognostic factors. All experimental statistical analyses were carried out using SPSS 22.0 statistical software package (SPSS Inc, Chicago, IL, USA). Statistical significance was set at a two-sided alpha level of 0.05. Graphs of biological experiments were drawn using GraphPad Prism 7 (GraphPad Software Inc, La Jolla, CA, USA).

## Supplementary Material

Supplementary Figures and Table
