# Supplementary material for: Rh type C-glycoprotein functions as a novel tumor suppressor gene by inhibiting tumorigenicity and metastasis in head and neck squamous cell carcinoma
Source: Aging (Albany NY). 2019 Jun 6;11(11):3601–23. doi: 10.18632/aging.102000 (PMC6594797; doi:10.18632/aging.102000)
Supplement: Supplementary Figures and Table [file aging-11-102000-s001.pdf]

SUPPLEMENTARY MATERIALS

Supplementary Figures

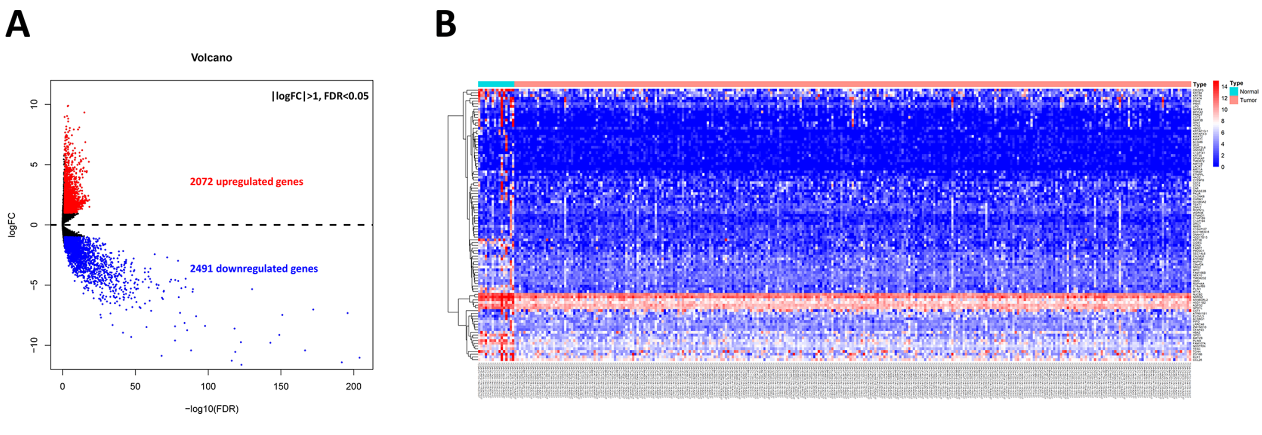

Supplementary Figure 1. DEGs identified in 299 HNSCC patients. (**A**) Volcano map of differently expressed genes between HNSCC tissues and normal tissues. (**B**) Heatmap of the top 100 DEGs according to the value of |logFC|.

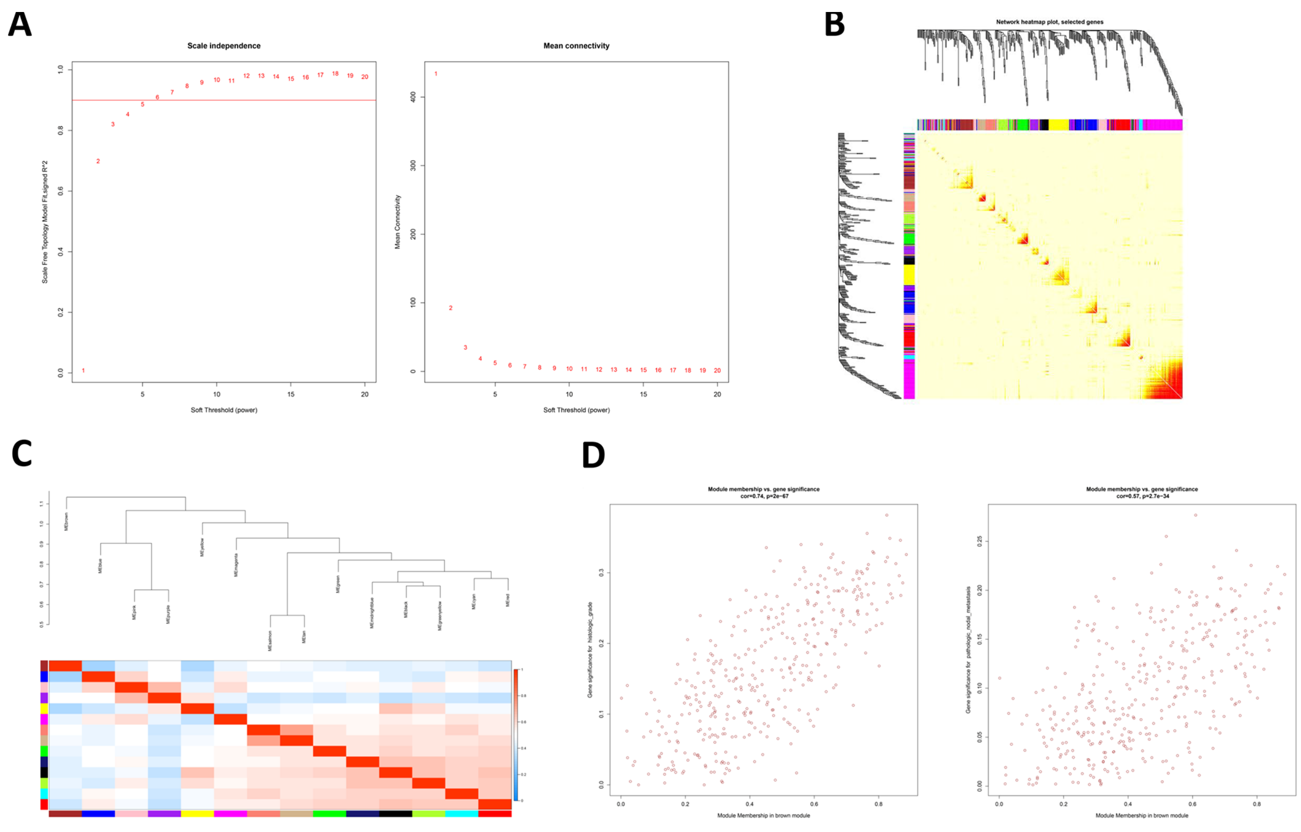

Supplementary Figure 2. Weighted gene co-expression network analysis. (**A**) Analysis of the scale-free fit index (left panel) and the mean connectivity (right panel) for various soft-thresholding powers (β). (**B**) A heatmap plot of 400 genes in the co-expression network was selected at random. The intensity of the red color indicates the strength of the correlation between pairs of modules on a linear scale. (**C**) Relationships among 14 modules. (**D**) A scatter plot of gene significance for histologic grade and pathologic nodal metastasis versus the module membership in the brown module.

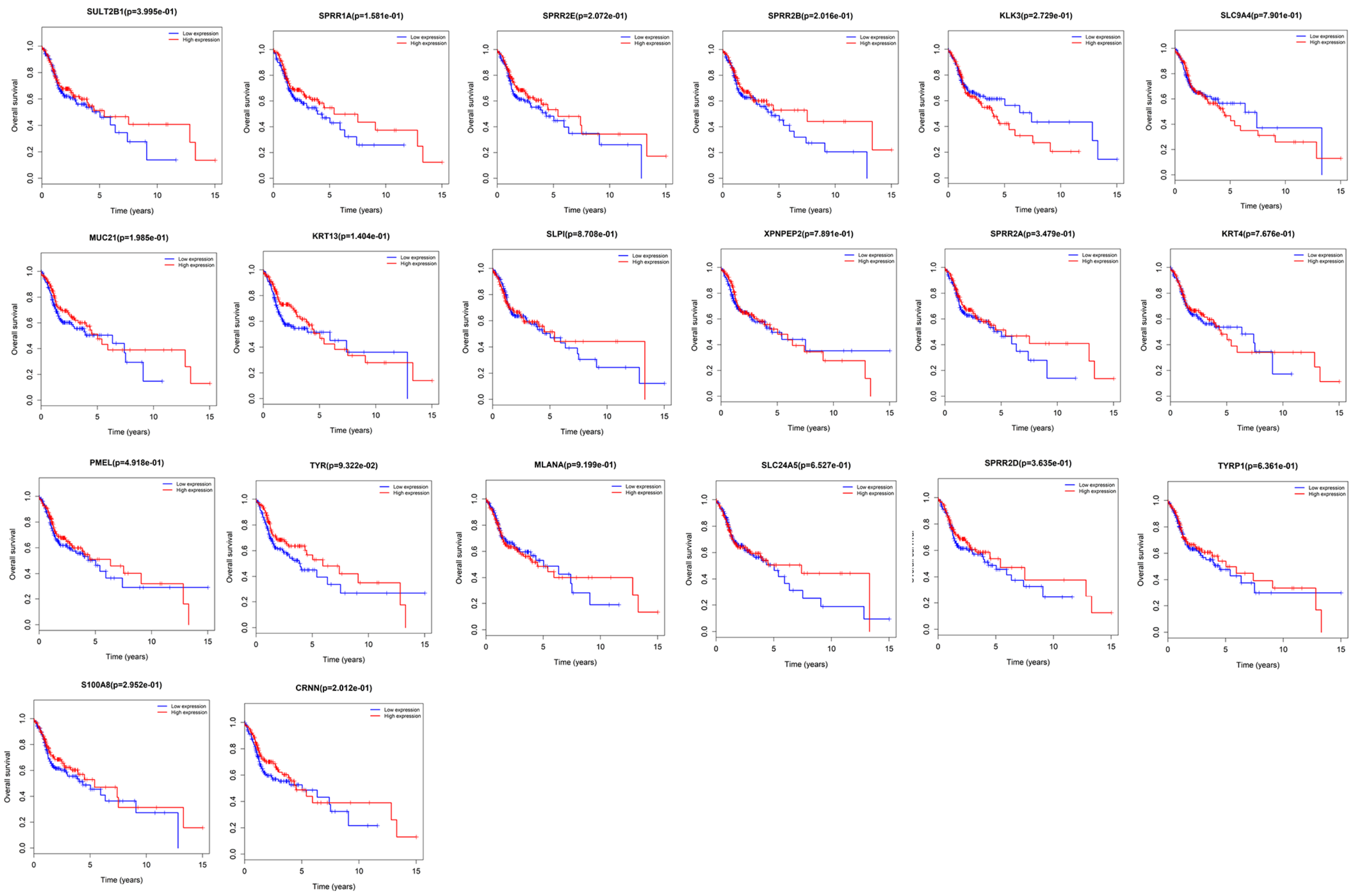

Supplementary Figure 3. Overall survival analyses on 20 hub genes in the 299 HNSCC patients. Survival curves for patients in different groups. Red lines represent high expression of hub genes, while blue lines represent low expression of hub genes

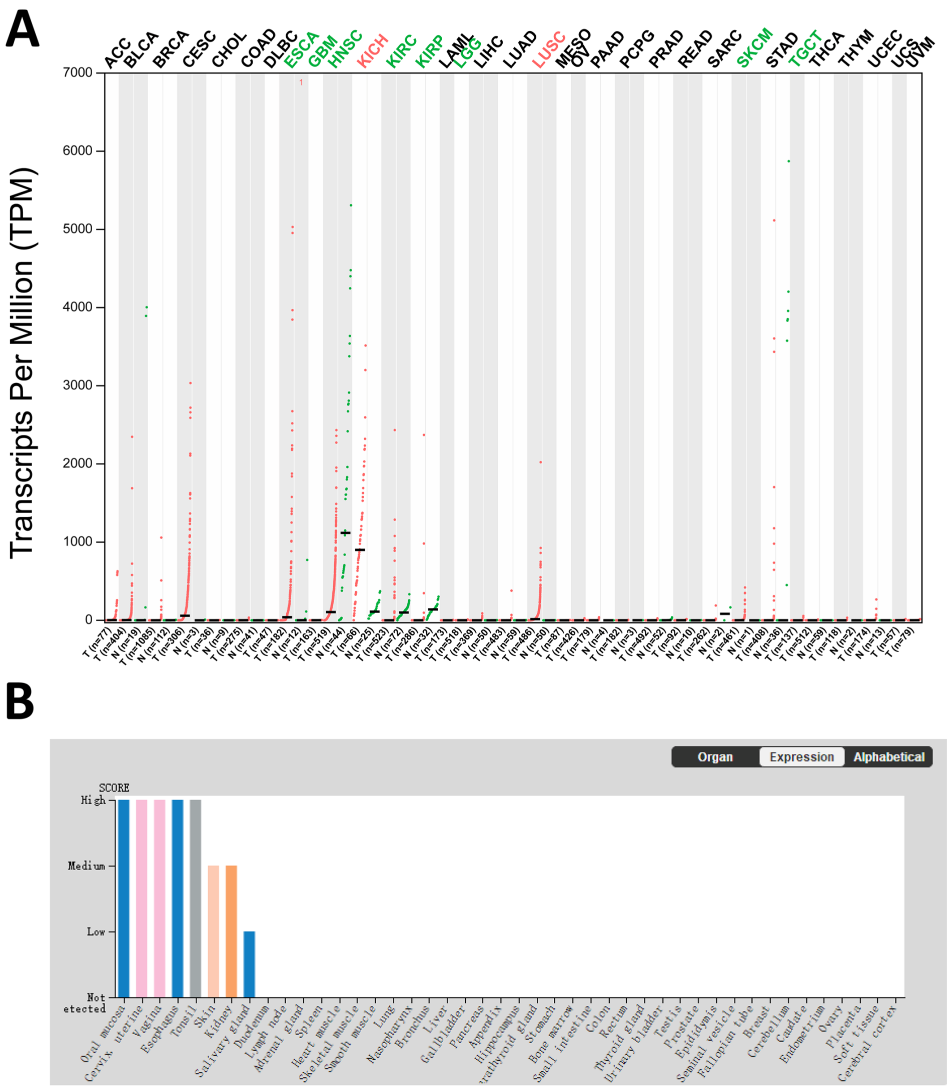

Supplementary Figure 4. An overview of RHCG expression profiling in tumor tissues and normal tissues for various cancers in TCGA database (**A**) and RHCG expression in normal tissues of different organs ranked by RHCG expression level (**B**).

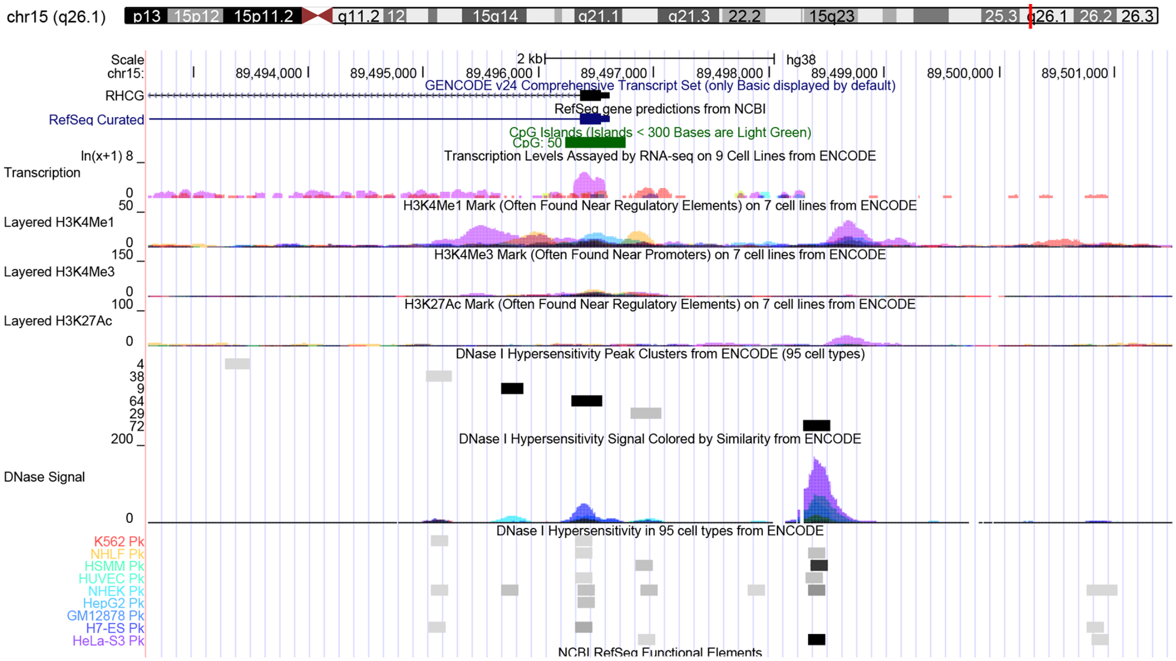

Supplementary Figure 5. Annotation of RHCG in UCSC database.

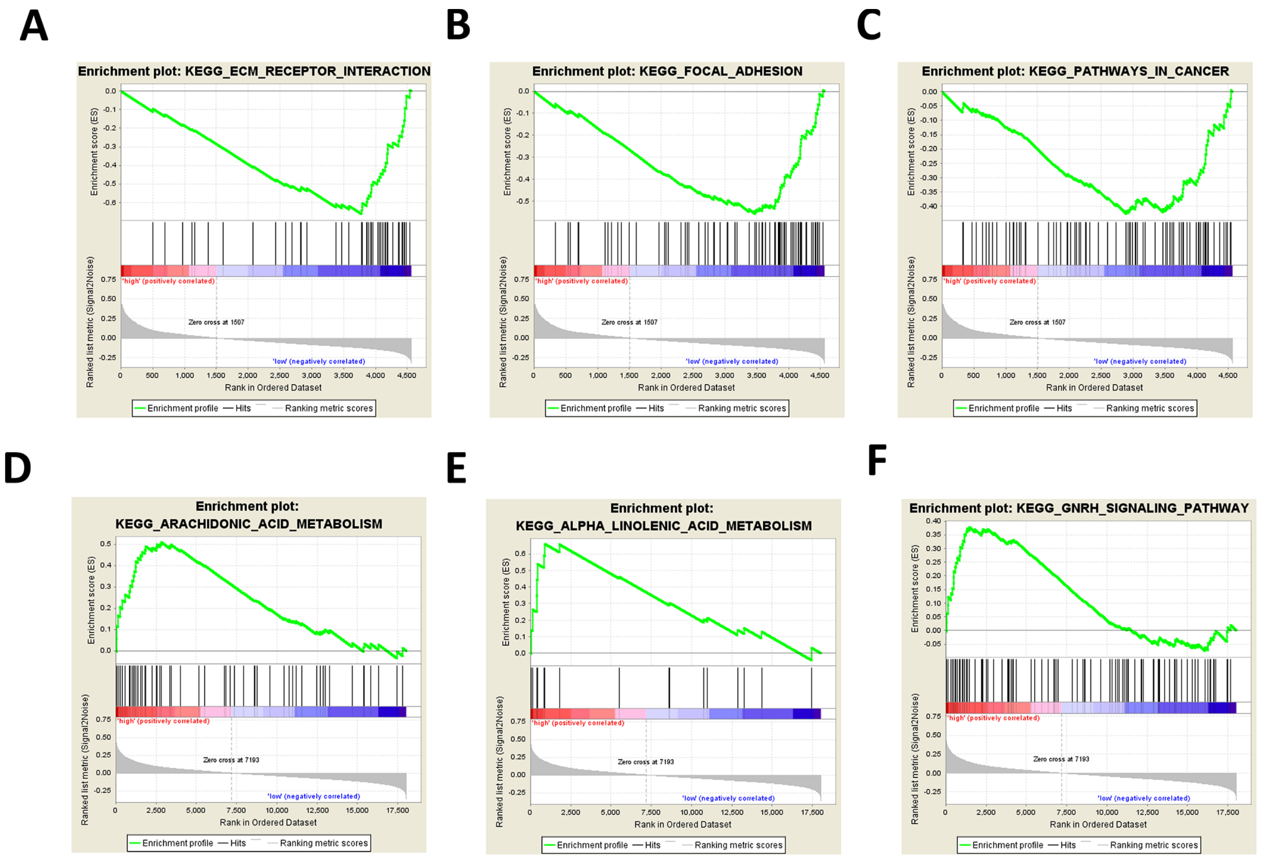

Supplementary Figure 6. Gene set enrichment analysis (GSEA) of 299 HNSCC patients stratified by RHCG expression. (**A**–**C**) Three functional gene sets of KEGG pathways enriched in HNSCC samples with RHCG downregulated. (**D**–**F**) Three functional gene sets of KEGG pathways enriched in HNSCC samples with RHCG upregulated.

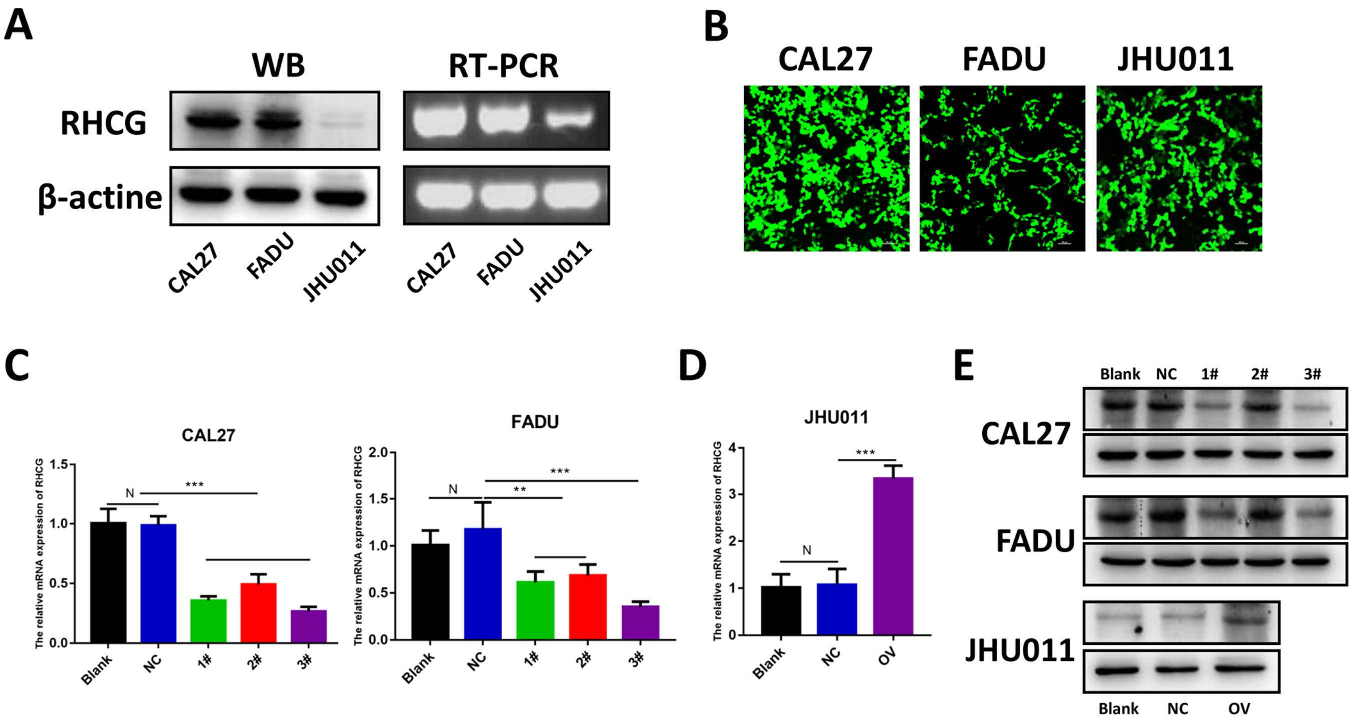

Supplementary Figure 7. Knockdown or overexpression of RHCG in HNSCC cell lines. (**A**) Protein and mRNA levels of RHCG were detected in CAL27, FADU and JHU011 cells without transfection; (**B**) Representative fluorescence images for transfection in CAL27, FADU and JHU011 cells. (**C**) mRNA levels of RHCG were detected by qPCR assays in CAL27 and FADU cells without transfection or transfected with NC or transfected with sh-RHCG 1#, sh-RHCG 2# and sh-RHCG3#. (**D**) mRNA levels of RHCG were detected by qPCR assays in JHU011 cells without transfection or transfected with NC and pcDNA-RHCG-transfected JHU011 cells (**E**) Protein levels of RHCG were detected by western blot assays in CAL27, FADU and JHU011 cells. β-actin served as a loading control. Data are presented as the mean ± SD of three independent experiments. *p < 0.05, **p < 0.01, ***p < 0.001, compared with the NC group.

Supplementary Table

Supplementary Table 1. The sequences of sh-RHCG1#, sh-RHCG2# and sh-RHCG3#.

NO.	5′	STEM	Loop	STEM	3′
RHCG - RNAi(1)-a	GATCC	GCTTACCTCGGTGGCAATATC	CTTCCTGTCAGA	GATATTGCCACCGAGGTAAGC	TTTTTG
RHCG - RNAi(1)-b	AATTCAAAAA	GCTTACCTCGGTGGCAATATC	TCTGACAGGAAG	GATATTGCCACCGAGGTAAGC	G
RHCG - RNAi(2)-a	GATCC	GCTTTGAGGATGCGGTCTACT	CTTCCTGTCAGA	AGTAGACCGCATCCTCAAAGC	TTTTTG
RHCG - RNAi(2)-b	AATTCAAAAA	GCTTTGAGGATGCGGTCTACT	TCTGACAGGAAG	AGTAGACCGCATCCTCAAAGC	G
RHCG - RNAi(3)-a	GATCC	GCAGGTGATTATGGTGATTCT	CTTCCTGTCAGA	AGAATCACCATAATCACCTGC	TTTTTG
RHCG - RNAi(3)-b	AATTCAAAAA	GCAGGTGATTATGGTGATTCT	TCTGACAGGAAG	AGAATCACCATAATCACCTGC	G
